# Imaging of Tumor Hypoxia With Radionuclide-Labeled Tracers for PET

**DOI:** 10.3389/fonc.2021.731503

**Published:** 2021-09-07

**Authors:** Yuan Huang, Junying Fan, Yi Li, Shaozhi Fu, Yue Chen, Jingbo Wu

**Affiliations:** ^1^Department of Oncology, The Affiliated Hospital of Southwest Medical University, Luzhou, China; ^2^Department of Oncology, Academician (Expert) Workstation of Sichuan Province, Luzhou, China; ^3^Nuclear Medicine and Molecular Imaging key Laboratory of Sichuan Province, Department of Nuclear Medicine, The Affiliated Hospital of Southwest Medical University, Luzhou, China

**Keywords:** positron emission tomography (PET), cancer, hypoxia, imaging, radiotracer

## Abstract

The hypoxic state in a solid tumor refers to the internal hypoxic environment that appears as the tumor volume increases (the maximum radius exceeds 180-200 microns). This state can promote angiogenesis, destroy the balance of the cell’s internal environment, and lead to resistance to radiotherapy and chemotherapy, as well as poor prognostic factors such as metastasis and recurrence. Therefore, accurate quantification, mapping, and monitoring of hypoxia, targeted therapy, and improvement of tumor hypoxia are of great significance for tumor treatment and improving patient survival. Despite many years of development, PET-based hypoxia imaging is still the most widely used evaluation method. This article provides a comprehensive overview of tumor hypoxia imaging using radionuclide-labeled PET tracers. We introduced the mechanism of tumor hypoxia and the reasons leading to the poor prognosis, and more comprehensively included the past, recent and ongoing studies of PET radiotracers for tumor hypoxia imaging. At the same time, the advantages and disadvantages of mainstream methods for detecting tumor hypoxia are summarized.

## Introduction

### Background

The tumor vascular system is the main factor determining the internal microenvironment of a tumor during its growth and is responsible for the exchange of oxygen, metabolites, and energy information between blood and tissue fluids. However, the vasculature of tumors differs from that of normal tissues. This structural difference increases the diffusion distance between the supplying vessels and tumor cells, forming a diffusion gradient and resulting in insufficient oxygen supply to the tumor cells that are away from the vessels, eventually leading to tissue hypoxia (1). As the volume of solid tumors continues to increase, especially when larger than 180–200 microns, coupled with the uncontrolled growth of tumor cells that consume high levels of oxygen and nutrients, tumor tissues will have insufficient local tissue perfusion or oxygen supply and lack of nutrients, resulting in the production of a series of acidic wastes and leading to extracellular acidosis. Collectively, these events ultimately result in tumor cells having a hypoxic microenvironment (2).

**Figure 1:**
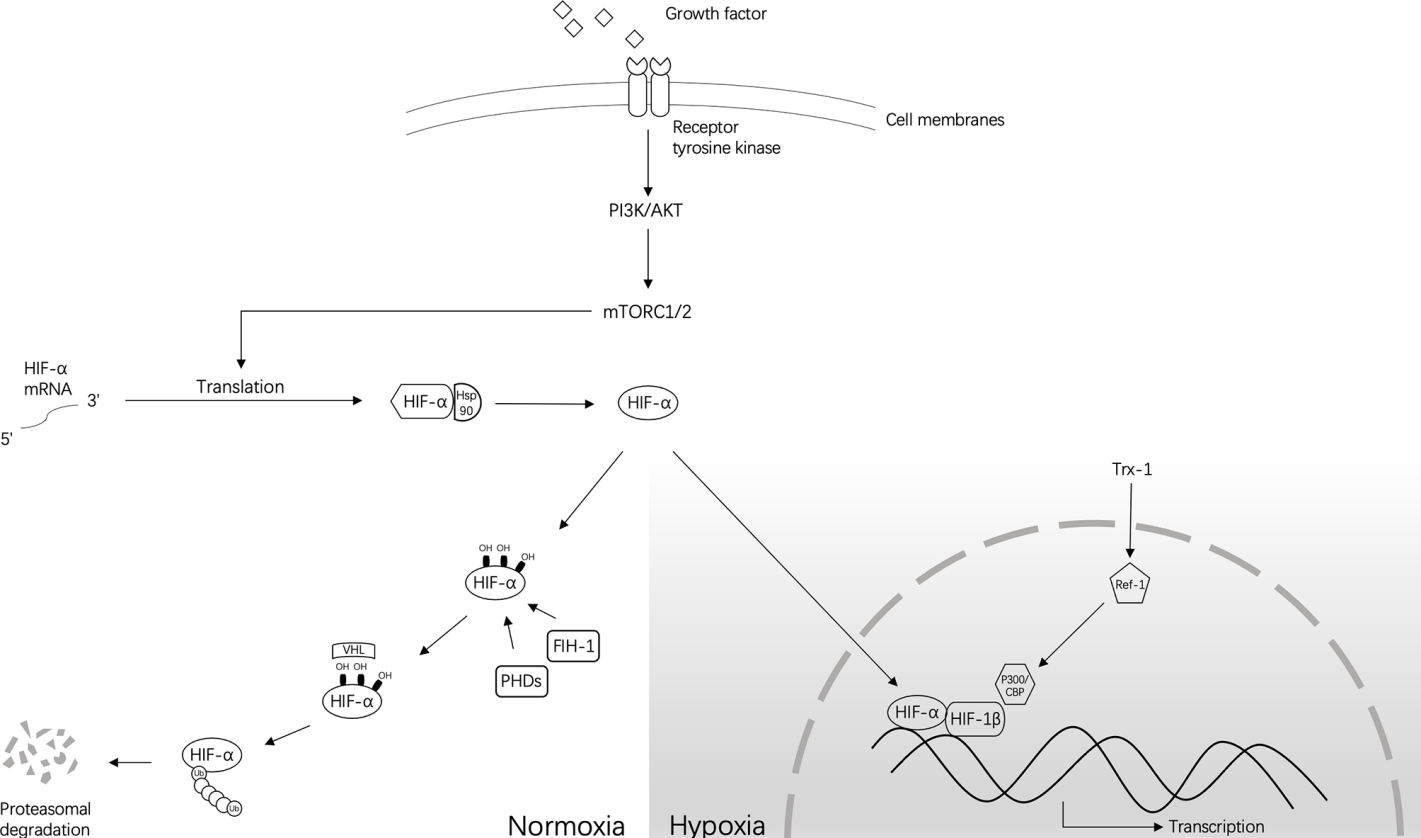
The approximate regulation of HIF-1 under normoxia and hypoxia. Normally, under normoxic conditions, HIF-α hydroxylation is mediated by prolyl 4-hydroxylase (PHD) and factors that inhibit HIF-1 (FIH-1). This process occurs at conserved residues. Hydroxylation of PHD causes instability of the HIF-α protein, and FIH-1 inhibits transcriptional activity by preventing the interaction of HIF-α and CBP/p300. Then, the ubiquitin-dependent process performed by the VHL complex mediates the subsequent degradation of HIF-α. However, under hypoxic conditions, PHD and FIH-1 are inactivated, leading to the stabilization of HIF-α and its translocation into the nucleus to dimerize with HIF-1β/ARNT and form HIF transcription factors. Without the involvement of FIH-1, HIF will transcribe a large number of target genes together with CBP/p300. Other small molecules that can regulate this process are also shown in this figure.

Hypoxia-inducible factor (HIF)-1 is a nuclear transcription factor that is widely present in mammalian cells. HIF-1 activates selected genes by transcription. These genes increase cell survival and proliferation to help cancer cells adapt to hypoxia by increasing angiogenesis, altering metabolism, degrading the extracellular matrix, inducing tumor cell dedifferentiation, and arresting the cell cycle (3–5). When the oxygen concentration is normal, HIF-1α is ubiquitinated and can be rapidly hydrolyzed by the ubiquitin-proteasome system. Therefore, HIF expression in normoxic cells is low (6). However, hypoxia prevents the ubiquitination of HIF-1α and induces an increase in the expression of HIF-1α (7). The transcriptional activity of HIF-1 also increases with the degree of hypoxia, resulting in activation of the p53 gene and expression of the cyclin-dependent kinase inhibitor (CKI) (8). Simultaneously, HIF-1α can also directly bind to the tumor suppressor gene p53 to promote p53-dependent cell apoptosis (9). In hypoxic conditions, the inhibition of prolyl hydroxylase stabilizes HIF-1α, followed by its binding to HIF-1β and transportation to the nucleus. The transcriptional activator protein p300/CBP is recruited through a transcriptional activation region of subunit a, and then interacts with the hypoxia response element (HRE), composed of the core sequence 5′-RCGTG-3′ of the promoter region of the target gene, such as carbonic anhydrase-IX (CA-IX). The downstream factors or enzymes are regulated and can then play a regulatory role (10) (**Figure 1**).

HIF can regulate vascular endothelial growth factor (VEGF), transforming growth factor (TGF), tumor necrosis factor (TNF), and angiopoietin-2 (ANG-2) receptor, among others (11), to induce the proliferation of vascular endothelial cells. While inducing new blood vessels, HIF also regulates the vascular system of the host for the metastasis of tumor cells (12). Thus, the tumor can not only obtain nutrients and oxygen from the host through the new tumor blood vessels, but can also continuously deliver metastatic cells to the host through these blood vessels, while continuing to grow and inducing blood-vessel formation in other parts of the body, ultimately leading to tumor invasion and migration (13, 14). It is noteworthy that the formation of new blood vessels may lead to reoxygenation of hypoxic areas. Using current standard radiotherapy, hypoxic cells can also gradually become reoxygenated (REOX) during the treatment process due to the killing of the radiosensitive oxygen-consuming cells near the capillaries, as the remaining cells can obtain more nutrients and oxygen (15). Activated HIF can also induce the formation of tumor cell stemness and screen out more aggressive cells.

Additionally, increasing experimental evidence suggests that CA-IX plays a direct role in many tumor phenotypic characteristics caused by hypoxia and acidosis, including increased local adhesion during cell proliferation, unstable cell contact, tumor interstitial crosstalk, and maintaining the stem cell phenotype, signal transduction, and other cancer-related phenomena (16). Importantly, CA-IX is not expressed in most normal tissues except in the stomach and gallbladder epithelium (17). However, it is usually overexpressed in tumors and supports tumor cell migration and invasion. Inhibiting the catalytic activity of CA-IX can significantly improve chemotherapeutic sensitivity or radiosensitivity. Similarly, inhibiting CA-IX can enhance the anti-angiogenic effect of anti-VEGF antibodies (18).

### Significance

**Figure 2:**
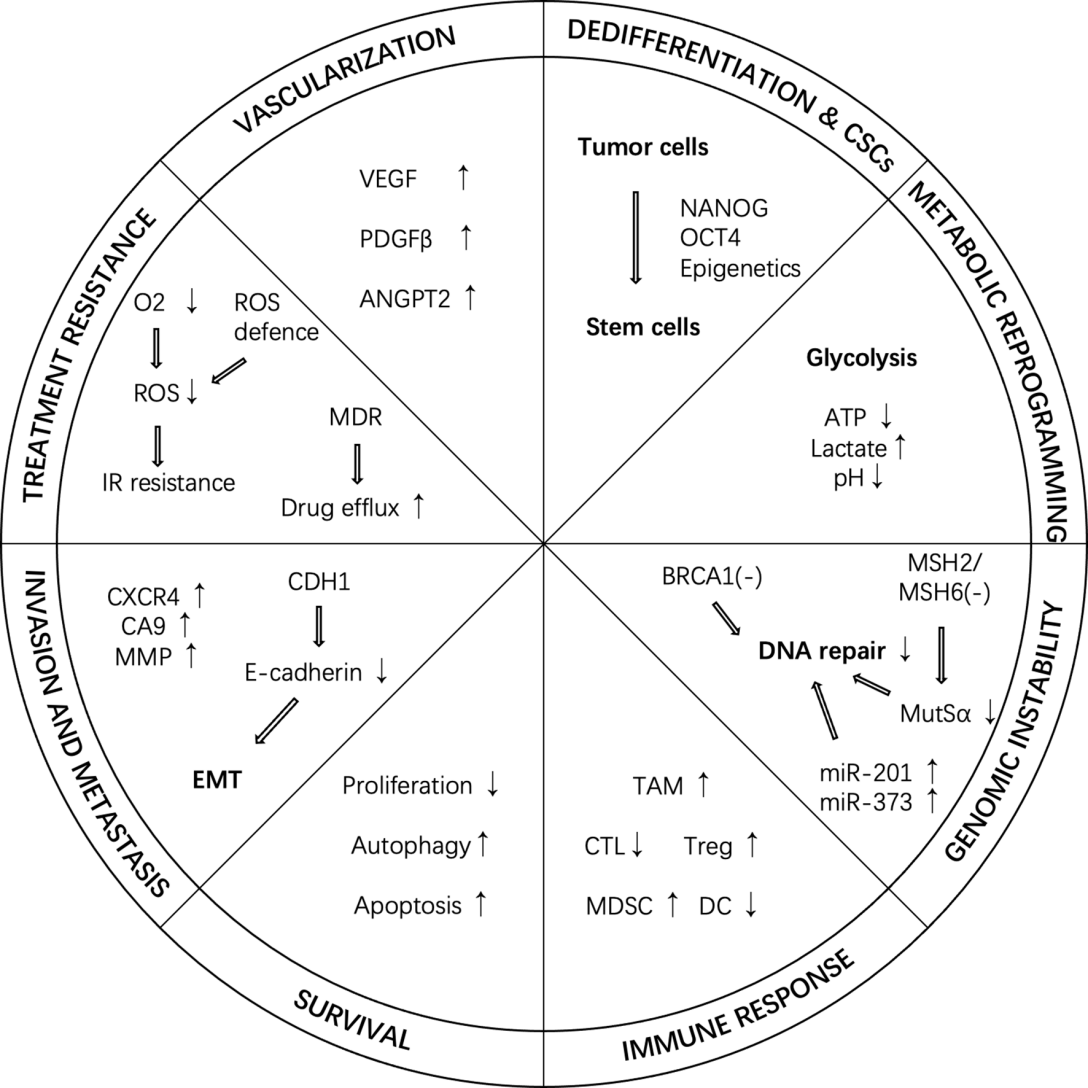
Cancer hallmarks regulated by HIF under hypoxic conditions. In hypoxic conditions, HIF can regulate a variety of cancer phenotypes, such as vascularization, dedifferentiation, metabolic reprogramming, genome instability, immune response, survival, invasion and metastasis, and treatment resistance. ANGPT2, angiopoietin 2; CTL, cytotoxic T-lymphocyte; CSC, cancer stem cell; DC, dendritic cell; IR, ionizing radiation; MDR, multi-drug resistance; MDSC, myeloid-derived suppressor cell; PDGF-β, platelet-derived growth factor-β; ROS, reactive oxygen species; TAM, tumor-associated macrophage; Treg, regulatory T-cell; VEGF, vascular endothelial growth factor.

Under hypoxic conditions, up-regulated HIF can regulate a variety of cancer phenotypes, causing various adverse changes in tumors and tissues (**Figure 2**).

Hypoxia can cause genetic damage. The rapid change in oxygen concentration produces free radicals, which act on tissue cells and cause DNA damage (19). Moreover, hypoxia may lead to the overexpression of tumor suppressor genes, pro-apoptotic factors, and anti-apoptotic factors to salvage damaged cells, but may make these cells polyploid. A study by Nelson et al. (20) shows that these cells have certain defects in their inherent apoptotic pathways, which are important signs of cancer and precancer.

The most important biological effect caused by hypoxia is resistance to radiotherapy and chemotherapy, which is directly related to disease prognosis. Molecular oxygen is very important for the sensitivity of radiotherapy. In conventional photon-based radiotherapy or light ion therapy, such as those using protons, oxygen has a high electron affinity and plays a central role in the fixation process (21, 22). Hypoxia reduces the ability of the drug to produce free oxygen radicals, resulting in hypoxic cells that are less sensitive to chemotherapy.

Another interesting phenomenon is that when the hypoxic state causes cell death, the migration of tumor-associated macrophages (TAM) to the hypoxic area is inhibited, thereby preventing TAM from removing dead and damaged cells. Other effects of TAM include inhibition of the immune response of the body to tumor cells and inhibition of the activation of adjacent macrophages. These phenomena promote angiogenesis and lymphangiogenesis, thereby promoting tumor growth, migration, and transformation (23, 24). Hypoxia-related TAM can upregulate HIF-1 and HIF-2 expression both *in vivo* and *in vitro* (25).

E-cadherin is an important intercellular adhesion molecule that mainly maintains the integrity and polarity of epithelial cells. Loss of E-cadherin is a sign of tumor epithelial-to-mesenchymal transition (EMT) in tumor cells and is related to hypoxia-induced HIF activation.

### Definition

There are considerable differences in the degree of hypoxia among different tumor types; thus, it is impossible to propose a definite value to define hypoxia. The currently recognized “gold standard” is to measure the distribution of the partial pressure of oxygen (pO2) using polarographic electrodes, but this method is invasive and greatly limits its use in tumor determination. As the oxygen requirement of tissues is met by blood, a method of responding to hypoxia by measuring the vascular supply was proposed. Cryophotometry or magnetic resonance imaging (MRI) to measure blood oxygen saturation, or MRI, computed tomography (CT), or positron emission tomography (PET) to measure tumor perfusion have been widely used (**Table 1**). However, vascular supply is not the only factor that causes hypoxia in cells and tissues; thus, monitoring of the vascular supply alone cannot completely determine hypoxia (50). Later, it was discovered that the expression of certain genes and proteins changes during hypoxia. Therefore, it was proposed that the state of hypoxia could be determined by measuring the levels of the endogenous markers. These principal markers include HIF-1, CA-IX, glucose transporter (GLUT)-1 and -3, and osteopontin (OPN). However, some reactions that are not related to hypoxia can also lead to upregulation of the endogenous markers. Some studies have reported that reactive oxygen species, cancer-specific mutations, and changes in signal transduction pathways can cause these changes even in normoxic conditions (51).

**Table 1:** Main methods of hypoxia assessment.

Technique	Mechanism	Advantage	Disadvantage
***Direct measurements***			
*Oxygen electrodes* (26–31) *(Eppendorf probes)*	Insert the electrode needle into the tumor for multiple measurements	Directly measure the partial pressure of oxygen at multiple points Closely related to the clinical outcomes of a small number of tumor types	Invasive and technically demanding. Suitable only for accessible tumors; risk of modifying oxygen concentration
*OxyLite* (32)	Continuous measurement at a single spot in a tumor.	Dynamic measurement (within several hours)	Invasive; not approved for clinical use
***Indirect measurements***			
*EPR* (33–35)	Paramagnetic probes altered by the surrounding paramagnetic O_2_ in electron relaxation procedure	No O_2_ consumption; dynamic measurement	Existing clinical equipment is unsuitable
*MRI–BOLD* (36–40)	Determines changes in the level of oxygenated hemoglobin	No O_2_ consumption; high temporal resolution	Easy to be disturbed; unsuitable for tissue hypoxia
*MRI Fluorine* (41)	Measures change in relaxivity of fluorinated probes in the presence of oxygen	No O_2_ consumption; dynamic measurement	Existing clinical equipment is unsuitable
*Redox-activated MRI contrast agents* (42–44)	Examines change in proton relaxivity due to reactive oxygen species with excitation of exogenous radicals	No O_2_ consumption; quantitative	Relatively low spatial resolution; requires a high concentration of contrast media
*PET*	Nitroimidazole: Radioisotope labeled nitroimidazole trapped in hypoxic cells (45–47) Cu-ATSM: Reduced metal complexes binding to macromolecules and trapped in hypoxic cells (48)	Noninvasive; high sensitivity; quantitative; assessment of the entire tumor volume; spatial mapping of hypoxia; serial assessment over time	Lack of a suitable trace; prone to false-positive results; ingested in normal tissues; unsatisfactory spatial resolution and tumor background ratio
*SPECT* (49)	Differential accumulation of SPECT tracers in areas of low oxygen concentration	Noninvasive; assessment of the entire tumor volume; spatial mapping of hypoxia; serial assessment over time	Resolution is limited; fewer agents than those available for PET; difficulties in quantifying hypoxia

However, the most widely used method for detecting hypoxia is based on PET/SPECT imaging. For example, foreign substances (imidazole drugs, cytarabine, or other substances) are labeled with radionuclides or fluorescent probes and used as tracers with specific chemical properties to target hypoxic areas within the tumor.

To summarize, pre-monitoring of hypoxic information before tumor diagnosis or treatment is extremely important in selecting accurate and effective individualized treatments and monitoring the efficacy and prognosis. As PET is more commonly used in a clinical setting for tumor diagnosis, in this review, we have focused and conducted a thorough evaluation on several methods that use markers in PET imaging to detect hypoxia. This study sheds light on the timely intensive treatment methodologies to overcome the resistance caused by hypoxia and prevent local recurrence.

## Main Method

Since 1981, misonidazole (Miso) was introduced as the first marker for the identification of tumor hypoxia based on molecular imaging. Miso was labeled with ^14^C for autoradiography to detect tumor hypoxia (52). Subsequently, two important PET tracer categories, 18F-labelled nitroimidazoles and Cu-labelled diacetyl-bis(N4-methylthiosemicarbazone) analogs, have been developed to specifically study the areas of tumor hypoxia (53).

### ^18^F

**Figure 3:**
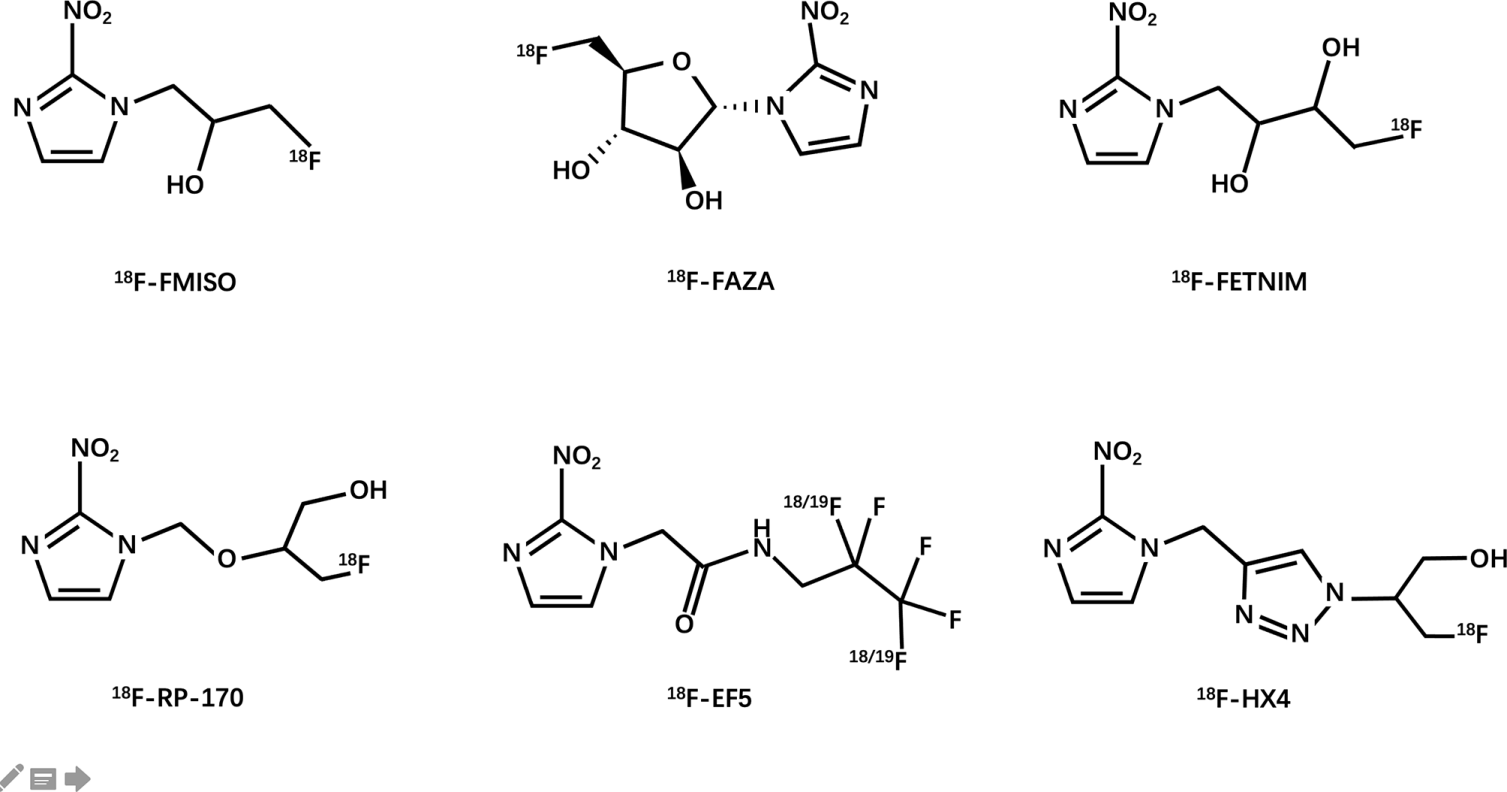
Structures of clinically used [^18^F]-labeled nitroimidazole compounds.

Janet Rasey and her colleagues at the University of Washington first proposed the noninvasive imaging of hypoxia by PET (54). They synthesized 18F-fluoromisonidazole and then verified its feasibility as a PET hypoxia tracer in several models of human tumors. Since then, researchers have extensively explored the possibility of using ^18^F-labeled compounds as hypoxia tracers (**Figure 3**).

#### Nitroimidazole

**Figure 4:**
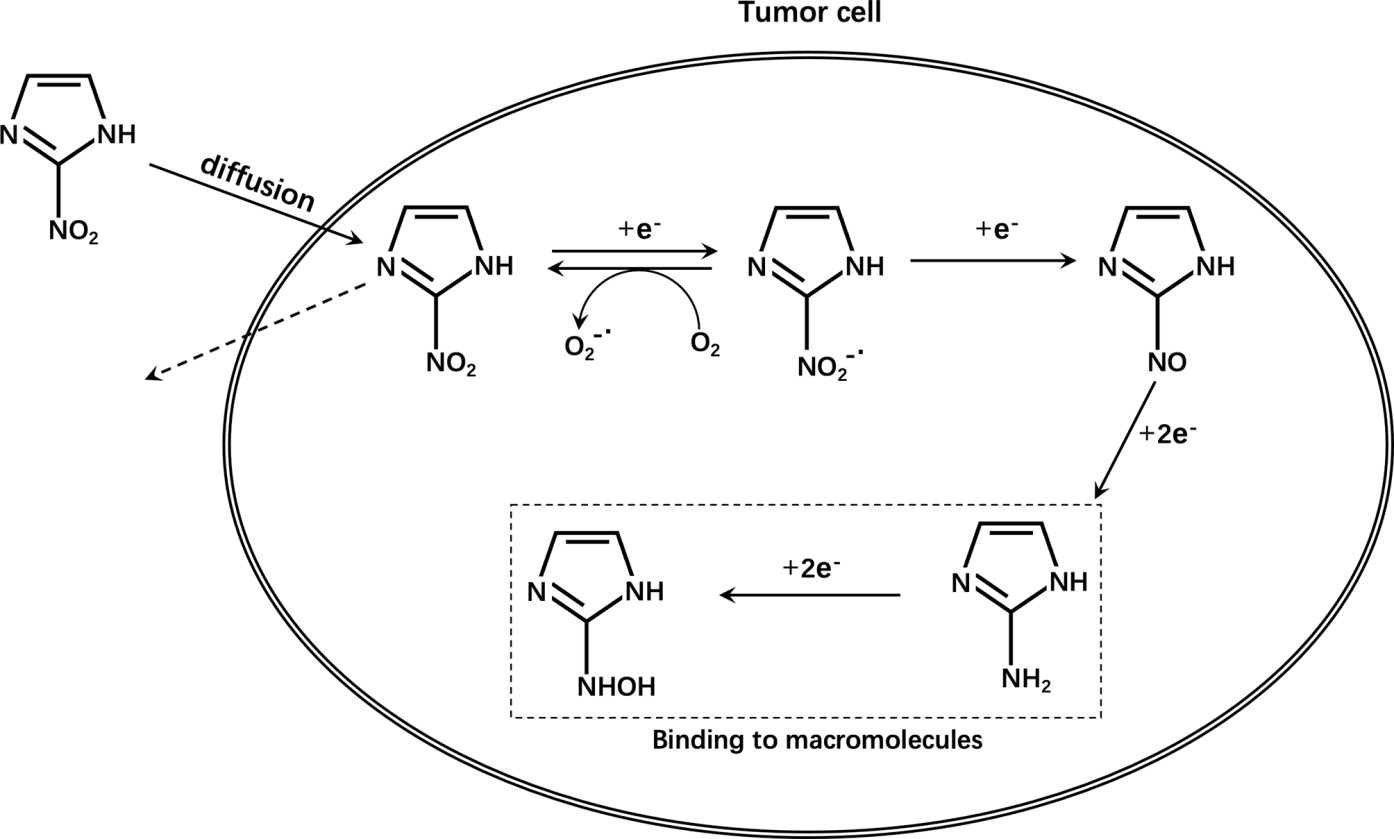
Schematic diagram of the mechanism of nitroimidazole compounds.

2-Nitroimidazole was originally developed as a radiosensitizer for hypoxic cells and proposed as a hypoxia marker in the 1970s (55). The reduction of nitroimidazole in cells is achieved by nitroreductases. The first one-electron reduction to a nitro group is reversible and the reduced substance is easily reoxidized by molecular oxygen (56). Therefore, substances reduced under aerobic conditions can be quickly reoxidized and can diffuse outside the cell without being retained in normal cells. However, under hypoxic conditions, the free nitro radicals are further reduced in cells, undergo protonation, combine with intracellular macromolecules, and are irreversibly retained in hypoxic cells (**Figure 4**). These radicals are not encountered in apoptotic or necrotic cells (57–59).However, the accumulation of nitroimidazole compounds in tumor cells is not only affected by hypoxia, but also by many other aspects, such as the expression level of multidrug resistance-associated protein 1 (MRP1) (60, 61). It is reported that tumor cells pretreated with MRP1 inhibitors have significantly higher radioactivity than cells not treated with inhibitors. At the same time, the SUV_mean_ ratio in tumors of mice in the treatment group is significantly higher than that of control mice *in vivo* PET studies. Therefore, it was found that MRP1 inhibitors can increase the accumulation of ^18^F-FMISO in hypoxic cells (62). In other words, during this type of chemotherapy, it may lead to an overestimation of tumor hypoxia.

#### ^18^F-FMISO

Over the years, several fluorinated nitroimidazole-based labels have been developed for PET imaging. Currently, the most commonly used PET hypoxia tracer used clinically and in research is ^18^F-fluoroimidazole (^18^F-FMISO) (63, 64). It is also the first drug to be clinically tested (65) and has been successfully used in glioma (66), breast cancer (67), head and neck tumors (68, 69), lung cancer (70), and other models to reflect hypoxia, and also used to stratify patients for radiotherapy. However, after intravenous injection, only medium contrast images are obtained owing to its high relative lipophilicity and slow penetration from the blood and passive diffusion, which results in a relatively low uptake (71). Compared with conventional imaging methods such as CT and MRI, ^18^F-FMISO PET has a lower resolution (5-7 mm) (72). These limitations of ^18^F-FMISO have led to the development of second-generation drugs with improved pharmacokinetic properties.

#### ^18^F-FAZA

The second-generation representative drug ^18^F-FAZA [1-(5-fluoro-5-deoxy-α-D-arabinofuranosyl)-2-nitroimidazole)] has better hydrophilicity and can enhance the signal-to-noise ratio. Its low lipophilicity leads to faster clearance of unbound drugs by the blood, which can improve hypoxia-normoxia contrast at an early point in time. In preclinical animal studies, the head-to-head comparison of ^18^F-FAZA, ^124^I-IAZA, and ^18^F-MISO showed that ^18^F-FAZA had faster vascular clearance than that of 18F-FMISO after 3 hours of injection. Recently, clinical studies have successfully evaluated the feasibility of ^18^F-FAZA in hypoxic imaging of brain glioma (73) and lymphoma (73); lung (74, 75), head and neck (76, 77), cervical spine (78), and cervical tumors (79); rhabdomyosarcoma (80); and rectal tumors (81). Generally speaking, 18-FAZA has better application values compared with ^18^F-FMISO.

#### ^18^F-FETNIM

^18^F-Fluoroerythronitromidazole (FETNIM) also has better hydrophilicity than ^18^F-FMISO, allowing for rapid renal clearance and low liver absorption, which can also explain the negative positive relationship between tumor blood flow and initial tumor ^18^F-FETNIM absorption (82). Clinical studies in esophageal cancer (83), head and neck tumors (84–86), and cervical cancer (87) have shown that the tumor-to-blood ratio calculated by imaging at 2 h pi is 1.4–2.48 within the range, and that high tissue uptake of FETNIM indicates reduced progression-free and overall survival. Although ^18^F-FETNIM can also be used in imaging studies to determine hypoxia in lung cancer (88, 89), some studies indicate that the uptake of ^18^F-FMISO in the tumor/non-tumor ratio is significantly higher than that of ^18^F-FETNIM. Therefore, whether ^18^F-FETNIM has a better application value than ^18^F-FMISO is still debatable (90).

#### ^18^F-RP-170

Another ^18^F-labeled 2-nitroimidazole compound, ^18^F-RP-170, has been used to study patients with glioma, and the average tpO2 in the high uptake area has been reported to be significantly lower than that in the low uptake area. In the high uptake area, a significant negative correlation between the standardized uptake value (SUV) and tpO2 has been noted, whereas the HIF-1α index in the high uptake area is significantly higher than that in the low uptake area. These findings are suggestive of the low oxygen selectivity of ^18^F-FRP-170 (91, 92). Studies on brain tumors (93) and lung cancer (94) show that the SUV in hypoxic tissue is higher than that in normal tissue. Compared with ^18^F-FMISO, the shorter time interval before scanning and improved hypoxic contrast may make it more suitable for research and use in clinical imaging.

#### ^18^F-EF5

^18^F-EF5 was studied as a PET tracer in 2001. Compared with many other hypoxia tracers, ^18^F-EF5 has a higher octanol-water partition coefficient, which enables higher cell membrane permeability and longer plasma half-life (95), which can improve the uniformity of tumor uptake and tracer distribution. In the study of patients with head and neck squamous cell carcinoma (HNSCC), the median tumor-to-muscle ^18^F-EF5 uptake ratio (T/M) was found to increase over time and reported as 1.38 (range, 1.1-3.2) after 3 h of tracer injection (96). The ability of ^18^F-EF5 to detect hypoxia is encouraging. It was also found in preclinical studies that ^18^F-EF5 PET could predict the response to graded radiotherapy in tumor models (97); however, its marking chemistry is more complicated by comparison.

#### ^18^F-HX4

A third-generation nitroimidazole tracer (^18^F-HX4) (98) has been developed in recent years. A 1,2,3-triazole moiety is introduced using simple click chemistry to make the compound more hydrophilic, which, at the same time, also increases its renal clearance (99). These characteristics of ^18^F-HX4 help in reducing the background signal faster, thereby improving the signal-to-noise ratio (98). Preliminary studies have shown that compared with ^18^F-FMISO and ^18^F-FAZA, ^18^F-HX4 has a higher maximum tumor-to-blood ratio with a half-life of about 3h (100). In addition, a piece of evidence that can prove that ^18^F-HX4 will accumulate in hypoxic areas is the observation of a strong and significant spatial relationship between the distribution of ^18^F-HX4 and pyridimidazole positivity and CA-IX positivity (98, 99, 101). In non-small cell lung cancer (NSCLC) (102), HNSCC (103, 104), and esophageal and pancreatic cancers (105), a clear correlation with ^18^FDG is observed, which is important however, better repeatability (106). Therefore, ^18^F-HX4 is a progressive next-generation tracer that can be used as a tool for monitoring treatment responses and radiotherapy planning.

### ^60-64^Cu

#### Copper (Cu)-diacetyl-bis (N4- methylthiosemicarbazone) (Cu-ATSM)

**Figure 5:**
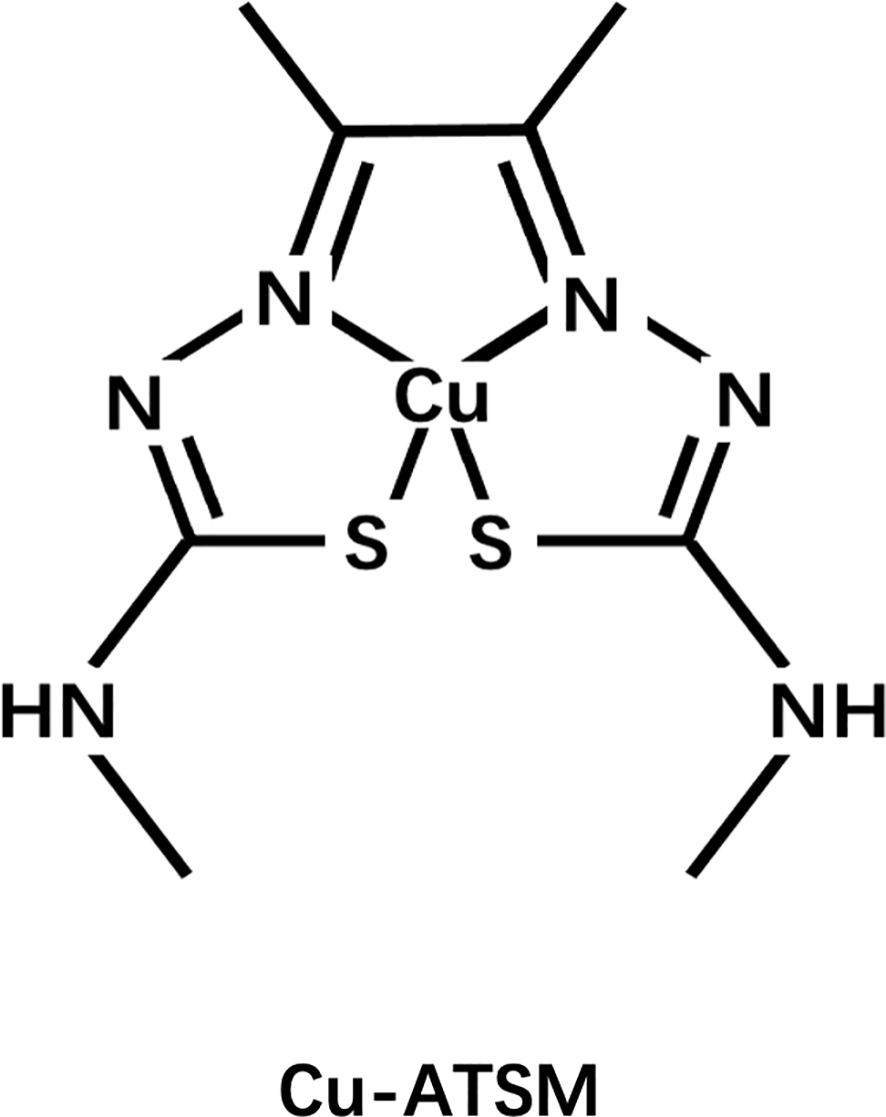
Structure of Cu-ATSM.

Another widely studied class of agents is the Cu complex with diacetyl-bis(N4-methylthiosemicarbazone) (ATSM) ligand, among which ATSM is the prototype (**Figure 5**). Fujibayashi et al. from Eukui Medical School in Japan (107) and Holland et al. from Washington University, St. Louis were the first to study the potential of these compounds in hypoxia imaging (108). Fujibayashi et al. used the ^60-64^Cu isotope to label some compounds such as ATSM and used the synthesized ^62^Cu-ATSM to study cardiac perfusion in a rat model. Their findings suggested that ^62^Cu-ATSM could accumulate during hypoxia. Similarly, their studies on ^64^Cu-ATSM, ^18^F-FMISO, and ^64^Cu-PTSM in EMT-6 tumor cells showed that ^64^Cu-ATSM and ^18^F-FMISO could differentially accumulate in tumors. In hypoxia, the accumulation was region specific and only ^64^Cu-PTSM was evenly distributed in the tumor (109). Cu-ATSM was first evaluated in humans as a radiodiagnostic agent for imaging lung cancer in the year 2000 (110), following successful preclinical trials (111–113).

**Figure 6:**
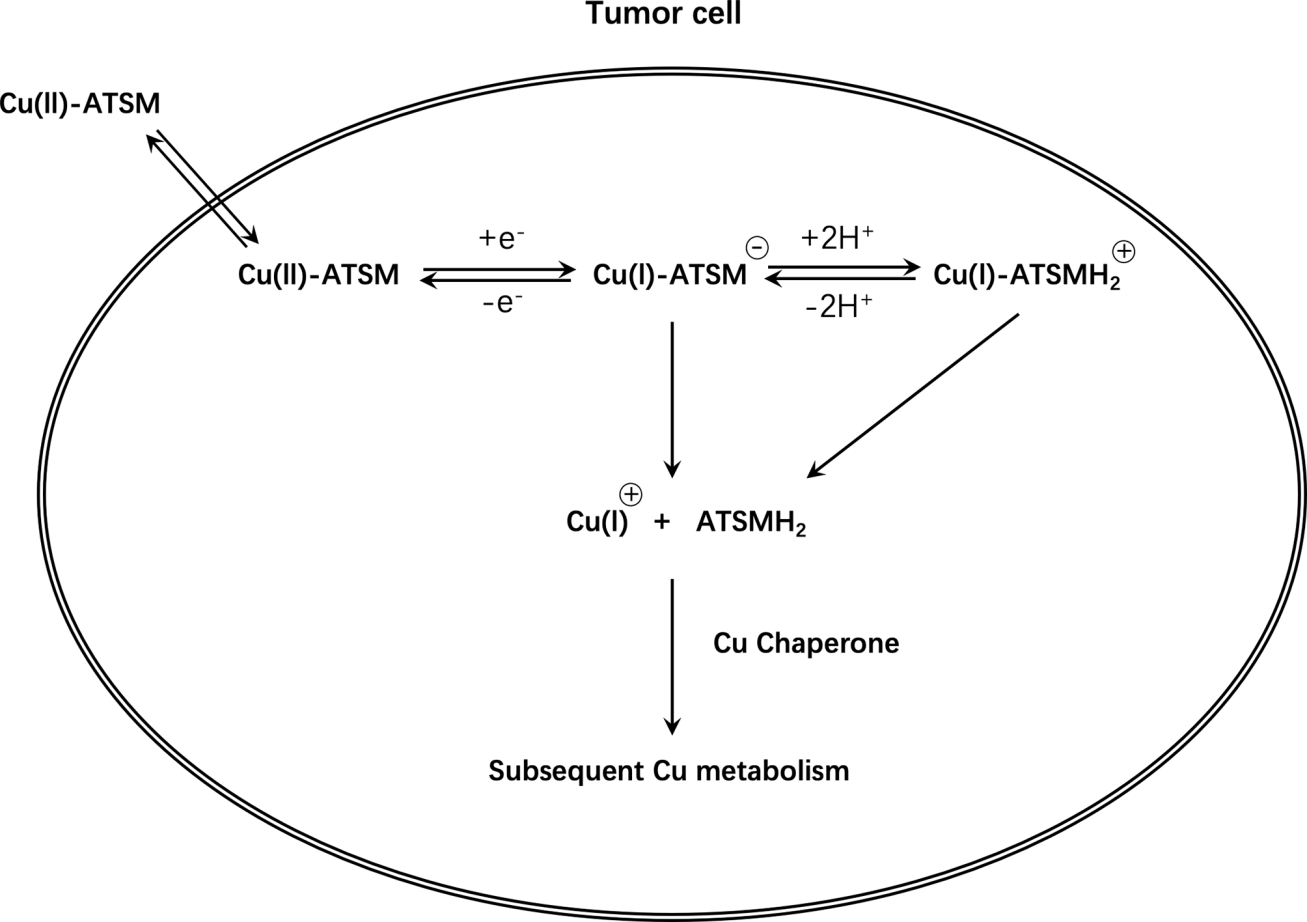
Schematic diagram of Cu-ATSM retention mechanism.

Unfortunately, the exact mechanism of Cu-ATSM hypoxia selectivity is unclear and controversial and has conflicting findings (114, 115). In conclusion, based on initial research, it is believed that Cu(II)-ATSM will diffuse rapidly into cells owing to its high membrane permeability and low redox potential, and will be metabolized by NADH/NADPH in the mitochondria that are dysfunctional due to hypoxia. Under normoxic conditions, Cu(I)-ATSM is reversibly oxidized to Cu(II)-ATSM and diffuses out of the cell. In contrast, under hypoxic conditions, the Cu(I)-ATSM complex is irreversibly retained in the cell. Since Cu(I)-ATSM is far less stable than Cu(II)-ATSM, under the mediation of pH and other conditions, copper dissociates from the complex to form ATSMH_2_, causing radioactive copper to remain in the cell (116, 117) (**Figure 6**).

Compared with ^18^F-FDG and fluorine-based hypoxic PET tracer, radiolabeled Cu-ATSM can specifically accumulate in the hypoxic areas of the tumor and form a certain difference. Moreover, Cu-ATSM exhibits better pharmacokinetics and signal-to-noise ratio, is not taken up by the bladder, and does not show any interference (118–122) (**Table 2**).

**Table 2:** Comparison of ^18^F-labeled nitroimidazoles and Cu-labeled diacetyl-bis(N4-methylthiosemicarbazone) highlighting the characteristics of the ideal hypoxia PET tracer.

	*^18^F-FMISO*	*^18^F-FAZA*	*^18^F-FETNIM*	*^18^F-RP-170*	*^18^F-EF5*	*^18^F-HX4*	*Cu-ATSM*
***1. Hypoxia specificity***	(123–125)	(123)	(123)	(91)	(95)	(123, 126)	(109, 127)
***2. Well-defined mechanism of retention***	(128)	(129)	(130)	(91)	(131)	(106)	(116, 117)
***3. Homogenous distribution and rapid clearance***	(123)	(123)	(123)	(132)	(96)	(106, 123, 126)	(113)
***4. Little dependency on factors that co-vary with hypoxia***	(133)	(134)	(135)	(92)	(96)	(136)	(137)
***5. Stability against non-hypoxic metabolism***	(138)			(92)	(95)	(123, 126)	(139)
***6. Suitable acquisition time***	(140)	(141)	(142)	(91)	(96)	(141)	(111, 112)
***7. Easy to synthesize and readily available***	(142)	(143)	(142)	(143)	(131)	(142, 144)	(116)
***8. Amenable dosimetry profile***	(123, 144)	(123)	(123, 144)	(91)	(145)	(123, 144)	(112)
***9. Repeatability in spatial uptake***	(146)	(147)	(148)	(91)	(149)	(150)	(111)
***10. Effective regardless of tumor type and stage***	(123)	(123)	(123)	(67, 142)	(151)	(123)	(136, 138, 152)

green (Yes= characteristics met); red (No= characteristics not met); blue = no consensus; blank = no data.

Detailed characteristics description: 1. Hypoxia is specifically retained in areas with low pO2 levels, but is not retained by normoxic or necrotic cells; 2. The retention mechanism of cells should be clearly defined and independent of cell type; 3. It should be hydrophilic enough to avoid membrane isolation, but also lipophilic enough to enter cells and allow uniform tissue distribution, and faster clearance from systemic circulation and normoxic tissues; 4. It’s pharmacokinetic characteristics and tissue distribution do not depend on parameters that may change with hypoxia, such as blood flow or pH; 5. It should have high stability against non-hypoxia specific metabolism in vivo; 6. The tissue kinetics should be suitable for imaging within the time frame allowed by clinical conditions; 7. It should be easy to synthesize and readily available; 8. It should have a amenable dosimetry profile; 9. It should be epeatable to allow detection of hypoxia and return to normal oxygen; 10. It should be effective for multiple tumor types (123).

Although its retention mechanism in hypoxic cells is still controversial, Cu-ATSM has been used as a PET tracer to study tumor hypoxia for head-and-neck (153, 154) lung (155, 156), cervical (157, 158), and rectal tumors (159), and gliomas (160). Studies have shown that Cu-ATSM has other benefits when used for hypoxia imaging, such as in the staging and detection of recurrent prostate cancer (120, 121); as an effective indicator to predict neoadjuvant chemoradiotherapy and survival rate of patients with rectal cancer (159); as an indicator of treatment response in head and neck cancer (161), and in brain tumors (162). In addition, due to its special radioactivity, many reports have highlighted its use as an effective radiotherapy agent. Since treatment is not within the scope of this review, it has not been discussed here (163, 164).

In summary, the above-mentioned studies show that the use of radiolabeled Cu-ATSM complex in PET examination is feasible and that other benefits to the treatment of patients are also possible (**Table 3**). Although the mechanism of action is unclear, it is still worth pursuing in-depth research and development on this compound.

**Table 3:** Partial summary of clinical imaging studies of hypoxia tracers.

Tumor type	*^18^F-FMISO*	*^18^F-FAZA*	*^18^F-FETNIM*	*^18^F-RP-170*	*^18^F-EF5*	*^18^F-HX4*	*Cu-ATSM*
**Brain**	(66, 165–167)	(73)		(92, 93)	-	-	-
**Head and Neck**	(68, 69, 168–175)	(73, 76, 77, 176–178)	(84–86, 179)		(96, 180)	(103, 104)	(153, 154, 181)
**Breast**	(67)						
**Sarcoma**	-						
**Lung**	(70, 182–187)	(74, 75)	(88, 89)	(94)		(102)	(155, 156, 188)
**Lymphoma**		(44)					
**Kidney**	-	-	-		-	-	-
**Liver**	-		-		-	-	-
**Colorectal**	-	(189)	-		-		(159)
**Bladder**	-	-	-		-	-	-
**Cervical**		(79)	(87)		-		(157, 158)
**Prostate**		(190)					-

green (Yes=good clinical data obtained); red (No=poor clinical data obtained); orange (Recommended=preclinical/metabolic data favorable); blue (Not recommended=preclinical/metabolic data unfavorable); blank=no data. Only relevant references in green and red categories are shown.

## Existing Research

### ^68^Ga

^68^Ga (t_1/2 =_ 68 _min_, 89% b+, Eb+max= 1.92 MeV, 11% EC) is a generator-produced radionuclide that offers excellent coordination chemistry with several bifunctional chelating agents and provides rapid radiolabeling over a range of pH (191–193). Therefore, ^68^Ga has been preferentially studied in hypoxic PET. For example, Sudhakara et al. developed ^68^Ga-labeled agents based on mono-, bis-, and tris-nitroimidazole conjugates with the chelating agent 1,4,7-triazacyclononane-1,4,7-tris[methyl(2-carboxyethyl)phosphinic acid] (TRAP), to obtain a series of ^68^Ga-labeled compounds. The radiochemical yields were extremely high and the uptake of tumor models in *in vivo* experiments revealed its remarkable ability to target hypoxic areas (194). Recently, Yoichi Shimizu et al. also confirmed that the ^68^Ga marker could help in the visualization of tumor tissues and hypoxic areas within 2 h of intravenous injection (195). In short, studies on lung (196) and colon cancers (197, 198) show that on the basis of the ease in production, ^68^Ga-labeled hypoxia tracers are comparable to traditional ^18^F-labeled tracers.

### ^124/125/131^I

Radioactive iodine is a commonly used radiotherapy agent for the diagnosis and treatment of thyroid diseases. The characteristics of its radiation make it well-sought after for radioimmunotherapy (199). Radioactive iodine isotopes play a role in the field of hypoxia imaging as they can be used to label various substrates that can target hypoxic regions. For example, ^123^I-IAZA is the most common radiotracer with a chemical structure similar to that of ^18^F-FAZA. ^124^I is a popular choice owing to its longer half-life. The distribution of ^131^I-IAZGP (200) is similar to that of pimonidazole, and has a similar CA-IX expression profile in colorectal cancer models. ^124^I-IAZGP (201) can be used to image liver tumors within 6 h (202); however, there are insufficient tumor-absorption studies that support the clinical role of ^124^I-IAZGP PET in patients with colorectal cancer and head and neck cancer. ^124^I-FIAU (203) can be used to evaluate the expression of HSV1-tkeGFP fusion gene that is associated with hypoxia. The area where ^125^I-IPOS accumulates is positively correlated with the HIF-1α-positive area (204). ^125^I-M75 specifically accumulates in colorectal cancer xenografts within 48 h of administration and delineates hypoxia by targeting CA-IX (205). However, several radioactive iodine isotopes have long half-lives. Although these isotopes play a unique role in radioimmunotherapy, their use as imaging agents should be carefully assessed.

### ^99m^Tc

The ^99m^Tc-labeled complex is one of the earliest non-nitroimidazole contrast agents used to determine hypoxia in cells. ^99m^Tc-labeled 2-nitroimidazole was also developed, with BMS181321 being the first. However, these agents are unstable and lipophilic, which limits their application (206). Subsequently, other tracers were developed to overcome these shortcomings (207). By choosing different cores and ligands, the tracer was designed to exhibit different characteristics to optimize the selection. The [^99m^Tc(CO)_3_ (H_2_O)]^+^ nucleus is well studied for the labeling of nitroimidazole compounds (208). Recently, more focus has been placed on the introduction of tumor-targeting moieties (e.g., RGD). The ^99m^Tc nitroimidazole complex is retained longer in hypoxic tumor cells.

### Others

In addition to the radionuclides discussed above, several others have been used for hypoxic PET. For example, ^111^In is a promising candidate for imaging hypoxic areas to study head and neck tumors (209), prostate cancer (210), and colorectal cancer (211). The nitroimidazole and gastrin-releasing peptide receptor (BB2R) conjugate was labeled with ^177^Lu (212); the CA-IX antibody G250-F(ab′)2 was labeled with ^89^Zr (213) and some promising results were obtained. Several nuclides are used in radiotherapy although their imaging effects are unsatisfactory or there are many factors worthy of improvement; however, these studies could help develop new ideas and research methodologies.

## Discussion and Conclusions

Assessing the relationship between tumor tissue and hypoxia has been a fascinating topic in cancer research for many years mainly due to its complex correlation with disease progression and treatment response. In clinical research, in addition to the use of oxygen electrodes for research, the use of exogenous probes such as FMISO/FAZA-PET, HX4, Cu-ATSM, and pimonidazole, as well as methods that target endogenous markers, such as CA-IX and HIF-1, are the mainstay to determine hypoxia (214). This review summarizes the progress of radionuclide-labeled PET tracers in hypoxia imaging. Each agent has its advantages and disadvantages; thus, it can only be used in a relatively limited range. Although some tracers have demonstrated the feasibility of their use in PET imaging hypoxia in several tumor entities in a clinical setting, so far, a perfect tracer with all desirable characteristics of an ideal PET hypoxia tracer has not been obtained.

One of the most important challenges in a clinical setting is the reproducibility of hypoxia imaging. The definition of the hypoxic area during measurement, selection of hypoxia-normoxia threshold, time variability of PO_2_ levels in tumors during continuous measurement, heterogeneity within and between tumors of the same patient, and factors such as receiving treatment affect the reproducibility of scanning (215).

Blood perfusion is one of the obstacles that limits the use of traditional hypoxia tracers in the tumor microenvironment. Insufficient perfusion limits the effective delivery of the tracer to tissues, affects tracer accumulation in normal or tumor tissues, and leads to poor imaging effects or contradictory findings. Therefore, the special relationship between perfusion and hypoxia requires further clinical research for the evaluation of hypoxia-perfusion patterns in different tumor types as well as to elucidate their relationship with clinical outcomes. With the recent advent of PET-MRI scanners, multiple imaging models and multifunctional tools that provide perfusion information and increase the accuracy of hypoxia measurement and provide complementary information with higher predictive values will soon become possible.

Many problems need to be solved urgently to obtain the ideal and perfect tracer. Defining a relatively standardized hypoxia threshold or observation index, comparing various tracers to study different tumor types, and screening a more optimized imaging plan may be suitable approaches to achieve this goal.

In recent years, the research and application of nanotechnology in tumors has resulted in a very valuable tool, which aims to provide various possible solutions to address the shortcomings of current imaging agents. Nanocarriers have better permeability and retention in solid tumor tissues; thus, they can promote molecular delivery to overcome the poor vascular system in tumor tissues. Nanocarriers can also be used to carry materials with different targeting properties, or can be used in imaging systems with multiple modes to improve their biocompatibility (by reducing cytotoxicity) and add other functions to improve the accuracy and utility of the tracer (216).

Owing to the increasing clinical demand for the assessment of hypoxia, imaging for tumor hypoxia is of great significance for tumor diagnosis, prognosis assessment, and planning treatment strategies. Due to hypoxia-induced malignant progression of tumor cells and their resistance to radiotherapy and chemotherapy, several ongoing clinical trials are focusing on adjusting the radiotherapy regimen according to the degree of oxygenation of the tumor and assessing the benefits. Simultaneously, radiosensitizers and hypoxia activation studies in medicine are also underway. Thus, the goal of researchers who are studying hypoxia is to change clinical outcomes rather than just provide prognostic information.

## Author Contributions

All authors listed have made a substantial, direct, and intellectual contribution to the work, and approved it for publication.

## Conflict of Interest

Authors SF, YC, JW was employed by company Academician (Expert) Workstation of Sichuan Province.

The remaining authors declare that the research was conducted in the absence of any commercial or financial relationships that could be construed as a potential conflict of interest.

## Publisher’s Note

All claims expressed in this article are solely those of the authors and do not necessarily represent those of their affiliated organizations, or those of the publisher, the editors and the reviewers. Any product that may be evaluated in this article, or claim that may be made by its manufacturer, is not guaranteed or endorsed by the publisher.

## Abbreviations

ANG-2, angiopoietin-2; ANGPT2, angiopoietin 2; BOLD, blood oxygen level-dependent; CAIX, carbonic anhydrase IX; CKI, cyclin-dependent kinase inhibitor; CT, computed tomography; CTL, cytotoxic T-lymphocyte; CSC, cancer stem cell; DC, dendritic cell; EMT, epithelial-to-mesenchymal transition; EPR, electron paramagnetic resonance; FIH-1, factor inhibiting HIF-1; GLUT, glucose transporter; HIF, hypoxia-inducible factor; HNSCC, head and neck squamous cell carcinoma; HRE, hypoxia responsive element; Hsp, heat shock protein; IR, ionizing radiation; MDR, multi-drug resistance; MDSC, myeloid-derived suppressor cell; MRI, magnetic resonance imaging; NSCLC, non-small cell lung cancer; OPN, osteopontin; oxphos, oxidative phosphorylation; PDGF-β, platelet-derived growth factor-β; PEG, polyethylene glycol; PET, positron emission tomography; PHDs, prolyl-4-hydroxylases; PI3K, phosphoinositide 3-kinase; REOX, reoxygenated; ROS, reactive oxygen species; SPECT, single-photon emission computed tomography; SUV, standardized uptake value; TAM, tumor-associated macrophage; TGF,transforming growth factor; TNF,tumor necrosis factor; Treg, regulatory T-cell; VEGF, vascular endothelial growth factor; VHL, von Hippel Lindau.
